# Developing Chinese university students’ academic literacies in English language classrooms via a production-oriented approach: an action research perspective

**DOI:** 10.3389/fpsyg.2023.1189555

**Published:** 2023-08-31

**Authors:** Yajuan Gao, Hao Wang

**Affiliations:** School of Applied Foreign Languages, Zhejiang International Studies University, Hangzhou, China

**Keywords:** university students, academic literacies, language competence, production-oriented approach, action research

## Abstract

Developing academic literacies is becoming an important research topic in TESOL and has received increasing scholarly attention. One of the difficulties in developing university students’ academic literacy in English language speaking is the lack of an authentic English language learning environment and the way to help students more effectively develop their language competence. To address this issue, this study seeks to explore the academic literacy development of 24 non-English major students in an 8-week-long integrated English class at a Chinese university from late 2022 to early 2023. Based on a production-oriented approach (POA) action research design, this study follows the typical framework of driving, enabling, and assessing phases to chain together the teaching and learning actions. Quantitative data from questionnaires were analyzed via SPSS software, whereas thematic analysis and content analysis were adopted in examining the qualitative data including classroom observations, teacher reflection journals, and semi-structured interviews. Based on our data analysis, this study finds that POA can have a positive effect on improving students’ participation, performance, and task completion, which improves their academic literacies. Implications on how POA helps in facilitating EFL students’ academic literacies development are discussed along with the acquisition model and future research directions like students’ identity issues and teachers’ perceptions of pedagogical approaches in teaching academic literacy within university settings.

## 1. Introduction

The National Medium- and Long-term Educational Reform and Development Program (2010–2020) in China points out that “improving teaching quality is the core task of the development of higher education,” which largely relies on high-quality foreign language education for university students. Therefore, the objectives in foreign language teaching are shifting from being able to obtain high test scores to using English in communicative ways, cultivating one’s cross-cultural awareness, developing students’ autonomous learning ability, and developing humanistic spirit and critical thinking skills (The National Advisory Committee on Foreign Languages Teaching in Higher Education, 2020). Over the years, one major problem that has been neglected in English classroom instruction in China is the separation of language learning and language teaching (Wen, 2015a). We believe that a lack of effective language teaching methods might be the main reason affecting the realization of the above teaching goals and the overall achievements of high-quality language education in higher education.

Studies have shown that EFL students face significant challenges in developing their academic literacy skills (Correa, 2010; Nagao, 2017; Singh, 2019; Zhang W. J., 2020; Rodas and Colombo, 2021). Among them, one major challenge is the lack of proficiency in English, which hinders their ability to read and write academic texts in the English language. For instance, Jalilifar (2009) found that Iranian EFL students struggled with academic writing, particularly in using appropriate grammar and vocabulary. EFL students often come from educational backgrounds that differ significantly from Western academic cultures, which makes it difficult for them to understand and meet academic expectations. Studies also found that East Asian students tend to rely more on dialectical thinking than their Western counterparts to solve problems (Lun et al., 2010). To address these challenges, researchers have proposed various strategies to improve EFL students’ academic literacies. One effective strategy is the use of explicit instruction, which involves providing students with clear instructions and feedback on how to improve their academic literacy skills (Roksa et al., 2017). Another strategy is providing EFL students with opportunities to practice academic writing or speaking in a supportive environment, such as peer-review and editing activities (Hyland and Hamp-Lyons, 2002). This approach can help students develop their writing skills through feedback and collaboration. While academic literacy research is growing (e.g., Al-Faraj, 2021; Li, 2022), the topic of how to improve EFL students’ academic literacies in classrooms remains underexplored. Research has overlooked how language teachers could facilitate students’ academic literacy development. Since language teaching is a social practice, students are not simply knowledge receivers; their background knowledge should also be taken into consideration. Therefore, pedagogical approaches need to reflect an understanding of language as a humanly constructed meaning-making model of reality (Byrnes and Kord, 2001). Thus, we believe the Production-Oriented Approach (POA) has potential, especially in integrating SLA theories into the social, cultural, and historical contexts of foreign language education in China (Wen, 2015b). The POA abides by the principles of a learning-centered focus, learning-using integration, cultural communication, and key competence. It encourages continual support by creating a scenario paving the way for activities to expose the gap between the knowledge mastered and the present teaching goal to input-enabled activities and collaborative assessment of the students’ processed production. This study demonstrates the effectiveness of POA in developing Chinese university students’ academic literacy in an English language class.

## 2. Literature review

### 2.1. Academic literacies

The construct of academic literacies first emerged as a research topic 20 years ago. Over the past two decades, multiple theories have contributed to our understanding of academic literacies (Li, 2022). In chronological order, three distinctive models emerged in the field of academic literacies research, namely the study skills model, academic socialization model, and academic literacies model. The study skills model, which views academic literacies as skill oriented, is the earliest model. It takes a language-based perspective in focusing on language use in academic settings and the language competence required for academic study. The disciplinary-based approach resembles the academic socialization model. The academic literacies model is concerned with meaning-making, identity, power, and authority (Lea and Street, 2006), pointing to higher-order language socialization, disciplinary-specific language, as well as cognitive and social practices. Among the three models, both the academic socialization model and academic literacies model are guided by sociocultural theory. In sociocultural theory, academic literacies highlight the transformative functions of academic literacies. Academic discourse is a type of writing and speaking skill that is used in specific fields and is vital to understanding academic literacies in that people communicate with each other in their own discourse communities. The development of these discourse conventions is viewed as the individual’s appropriation of the shared cultural values and communicative repertoires within the disciplines (Li, 2022). Framed from a sociocultural perspective, the current study defines academic literacy as an integration of both language skills and competence that facilitate language learners’ academic development.

Research finds that developing academic literacy is a powerful tool for knowledge generation, communication, and transformation (Li, 2022). Academic literacy is an essential skill for students as it enables them to comprehend and critically analyze academic texts, conduct research, and communicate their ideas effectively. Several factors have been identified as influencing students’ academic literacies, including their prior knowledge, motivation, language proficiency, and socio-economic status. For example, studies show that students with higher levels of prior knowledge and motivation tend to be more proficient in academic literacy than those with lower levels of prior knowledge and motivation. Language proficiency is also crucial for academic literacy, as students who are not fluent in English may struggle to comprehend academic texts and communicate their ideas effectively (Grabe and Stoller, 2011). Finally, socio-economic status plays an important role in academic literacy; those from lower socio-economic backgrounds often face more barriers to developing academic literacy skills (Lam, 2009).

Research also suggests that the development of the advanced literacy skills can only be achieved via students’ active engagement in authentic and purposeful disciplinary learning activities, imbued with meaning, value, and emotions (Moje, 2008). Various approaches have been proposed for teaching academic literacies, including explicit instruction, genre-based approaches, and content-based instruction (Brinton et al., 1989). Explicit instruction involves direct teaching of academic literacy skills, such as reading comprehension, academic writing, and critical thinking, while genre-based approaches focus on teaching students the language and structures of specific academic genres, such as research articles or essays (Swales and Feak, 2004). Content-based instruction integrates academic literacies instruction into content-area courses, such as science or history, and aims to develop students’ academic literacy skills using authentic materials and tasks (Brinton et al., 1989).

### 2.2. Production-oriented approach

The Production-Oriented Approach (POA) was proposed in 2015 to explore local language teaching approaches in China. The POA adapts classic SLA pedagogical theories to the social, cultural, and historical contexts of English education in China and has its roots in the communicative approach (Wen, 2015b). The communicative language teaching approach emerged in the 1970s as a response to traditional grammar-based approaches. It emphasizes the importance of communication and meaningful interaction in language learning (Wen, 2019). The POA builds on this by emphasizing the development of practical language skills through hands-on, project-based learning.

**Figure 1:**
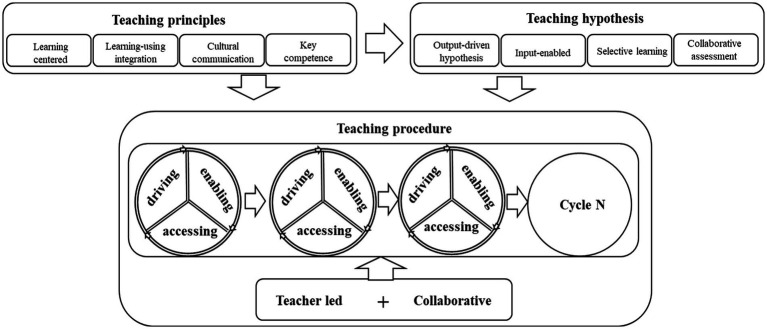
Theoretical framework of POA 3.0 (adapted from Wen, 2022).

Inspired by the output hypothesis (Swain, 1985), input hypothesis (Krashen, 1985), and interaction hypothesis (Long, 1983), the POA challenges the traditional understanding of “input materials are input” and “output (production) is output only”: it puts forward the idea that output also serves as the driving force for effective teaching and learning, especially for the intake of the input (Wen, 2015a). The POA challenges the text-centered Intensive Reading Approach (IRA) and advocates for a new teaching approach that engages students in language use while learning with a teacher’s mediation (Wen, 2015b, 2016). Influenced by sociocultural theory, the POA claims that with the guidance of an expert an individual can perform a range of abilities they cannot perform on their own; thus an individual has opportunities to develop new skills (Eun, 2017). Specifically, the POA builds on four hypotheses: the output-driven hypothesis, input-enabled hypothesis, selective learning hypothesis, and collaborative assessment hypothesis (Wen, 2018), seen in the theoretical framework of POA (Figure 1).

Since its inception, the production-oriented approach enjoys wide popularity among language teachers in China, particularly regarding its teaching principles, namely, the learning-centered principle (Wen, 2015a), learning-using integration principle, and whole person principle (Wen, 2016). Different from traditional approaches that favor teacher-centeredness or student-centeredness, the POA believes that all teaching activities should be designed to guarantee effective learning. And the learning-using integration principle means the input materials should directly serve the purpose of fulfilling productive tasks with new knowledge or skills. The third principle is the cultural communication principle, which echoes the educational goal, and the fourth principle is the key competence principle, as it is “teachable and measurable” (Wen, 2018).

In the context of foreign language teaching designed via the POA, students are involved in using the target language in communicative situations. This approach emphasizes practical and hands-on learning experiences and encourages students to apply their knowledge of the target language in real-world situations. The production-oriented approach is based on the idea that students learn best when they are actively engaged in the learning process and could apply their knowledge in a meaningful context. Several studies have explored the effectiveness of the production-oriented approach in foreign language teaching. For example, a study by Duan (2021) found that using project-based learning in the English language flipped classrooms could lead to significant improvement in students’ speaking and writing skills. However, some studies have also highlighted potential limitations of the production-oriented approach in foreign language teaching, such as the challenges to implement it in large classes and resource limitations (Bransford et al., 2000).

Based on the aforementioned literature, two research questions guided this study:

(i) How do we implement the POA in a university English language class?
(ii) In what ways could the POA develop Chinese university EFL students’ academic literacies?

## 3. Methodology

### 3.1. Context and participants

This study took place in a southeastern liberal-arts-based university in China. Students are enrolled into this university based on their college entrance examination scores and are grouped into different levels of English language classes depending on their placement test results. The researchers sought different ways to invite potential participants for this study via speaking in class, WeChat friend moments, and flyers in early November 2022. Questionnaires were distributed in the first researcher’s three classes, and 142 copies (77.6%) were collected. Based on the result of the pre-test, 24 non-English major students in total from three classes were studied in this research, with eight coming from each class.

Demographic information about participants was collected in the following areas: gender, age, home city, level of foreign language taught, number of years taught, and frequency of foreign language use.

As shown in Table 1, the participants are of similar ages because they are in their first 2 years of university, and they had on average 12–14 years of English learning experience. They claimed to have had on average 2–4 opportunities to communicate with foreigners in English outside of class in the previous year. All eight participants from each class are chosen by their result from the pretest, with two scoring above 80, two scoring above 70, two scoring above 60, and two scoring above 50. Their willingness to speak ranges from 6 to 8 out of 10, indicating a strong willingness to use the language learned in class.

**Table 1:** Participants’ demographic information.

	Class1	Class2	Class3
Gender	M5/F3	M2/F6	M4/F4
Age	18.3	18.4	19.3
Major	Science Education	Languages	Mixed
Home city	1/2 city tier:3	1/2 city tier:5	1/2 city tier:5
	3/4 city tier:5	3/4 city tier:3	3/4 city tier:3
Years of learning	12.7	12.9	13.6
Language use (times in previous year)	3.2	2.5	2.7
English proficiency	H2/M6/L2	H2/M6/L2	H2/M6/L2
Willingness to speak (out of 10)	7.0	7.9	6.1

### 3.2. Research method

This study follows an action research design, as it enables researchers to find effective solutions to problems in their teaching practices. Established by Kurt Lewin in the 1940s, Action research offers clear operation procedures so that researchers can follow. It has three parts, including identifying a problem or question, carrying out an action, and observing and reflecting on the outcome (Lewin, 1946). Action research seeks to engage the “complex dynamics involved” (Stringer, 2014, p. 6). In language teaching contexts, researchers have employed action research via continuing cycles of investigation to find effective solutions to problems in teaching and learning (Zhang H., 2020). Spanning 8 weeks aside from the winter break, this action research probes into the effectiveness of the POA in developing Chinese students’ academic literacies in English language classrooms.

### 3.3. Data collection and analysis

In this study, we used students’ test results, questionnaires, and interviews as the main data sources. In addition, we also used class observation classroom videos and journals to supplement data collection. Test results are students’ output grades in Cycle 1 and pre-tests and post-tests results in both Cycle 2 and Cycle 3. Questionnaires in the form of online surveys were sent to students’ smartphones at the beginning and end of the research project to extract the students’ knowledge of the teaching topic as well as their impression and sense of achievement. Interviews were conducted after each cycle to understand students’ feedback on the teaching procedures and content. Class observations were conducted by the first author and reviewed by the second author. Audio and video recordings were collected by a digital recorder and a camera in the classroom. Students were also asked to keep a journal that documents their experiences regarding the effectiveness of the POA in developing their academic literacies (Table 2).

**Table 2:** Data sources.

Data source	Duration/number	Analysis
Interview	6+ hrs.	Thematic analysis
Observation	4 wks.	Field notes
Journal	19,000+ wds	Content analysis
Audio + video	144 min	Content analysis
Questionnaire	2	Likert scale
Test results	1+ 2*2 = 5 tests	Graded on 10 scale by experts

Data from questionnaires and tests were sorted from a quantitative perspective and analyzed with SPSS and content analysis; qualitative data collected from interviews, assignments, and journals were analyzed by coding via thematic analysis. Respondents were asked to rate each item on a Likert-type scale from 1 (strongly disagree) to 5 (strongly agree) in terms of how much it contributes to effective foreign language learning.

According to the analysis of 142 questionnaires (with 117 valid responses), 82% of the respondents expressed an urgent need for improvement in English language proficiency. Additionally, 75% believed that the teaching methods they had previously received were not task-driven, resulting in a lack of practical application and negligence of learning-using integration. The respondents expressed a desire to enhance their academic proficiency in language expression in areas such as language skills (81%), scenarios (72%), and cultural background (69%).

## 4. Research design

The current study has four phases: identifying the problem, identify the research setting, planning and implementation, as well as reporting research findings.

### 4.1. Steps of current action research

This action research follows the 10 steps below:

(i) Identify the language teaching problem: Chinese students’ cultural unawareness when communicating with foreigners.
(ii) Literature review: reading journal articles relevant to the problem and discuss with colleagues in the same teaching group.
(iii) Identify the research questions:

- How do we implement the POA in a university English language class?
- In what ways could the POA develop Chinese university students’ academic literacies?

(iv) Further literature review: explore further readings on academic literacies and the production-oriented approach.
(v) Make action plans: based on the literature review and research questions, decide on the participants and data collection in the study.
(vi) Conduct a pilot study: based on the action plan, analyze and explain the data collected at this stage.
(vii) Nail down the action plan for the current study: with the findings from the pilot study and previous literature, nail down the action plan for the current study based on the real educational setting.
(viii) Implement the action plan: through questionnaires, class observations, students’ texts, students’ journals of learning experiences, interviews, and teachers’ reflection journals, researchers are to identify the challenges in the students’ production and analyze the gap between teaching and learning in the language teaching classroom as well ask the following questions: Is the designed activity is necessary to the teaching practice? Is it more effective or less effective if conducted strictly following the POA procedure of driving, enabling, and assessing?
(ix) Evaluate the implementation of the action: analyze the students’ productions and report the initial findings with the help of post interviews and reflect on the improvements and decide whether the result is a satisfying one: What are the crucial parts of the plan that have the most weight?
(x) Perfect the action plan and run a new cycle of implementation: based on step 9, decide whether further exploration is necessary or whether a new problem is detected and conduct a new cycle should be conducted.

Together, three cycles of teaching were conducted, the first cycle was done in a traditional teaching mode and the following two cycles were done via POA corresponding to the driving, enabling, and assessing phases. The students’ production of videos and texts were then carefully examined by four experts and then reviewed by the researchers.

### 4.2. Sampling

This study involves students from three English language classes and two English language teachers. To explore the effectiveness in each teaching cycle, subjects were the eight students in each class whose first production scores range from 50–60 (2 students), 60–70 (2 students), 70–80 (2 students), and 80–90 (2 students).

### 4.3. Rubrics of students’ production

Under the Common European Framework of Reference for Languages: Learning, Teaching, Assessment, this study adopts the standards for English language proficiency by Renmin University of China. The standards break down the assessment into five specific aspects of the students’ English-speaking competence: linguistic competence, sociolinguistic competence, pragmatic competence, strategic competence, and cultural awareness (RUC-SOPE in Renmin University, 2021).

In the current study, the researcher adopted a scale of 10% linguistic competence, 10% sociolinguistic competence, 20% pragmatic competence, 30% strategic competence, and 30% cultural awareness and invited four experts as panel members (C1\C2\C3\F) to review the files collected. Among the four experts, C1, C2, and C3 are experienced Chinese university English teachers majoring in linguistics, translation studies, and language assessment; F is an American middle school teacher, majoring in education. Therefore, an average score of score (X) = 1/4(C1 + C2 + C3 + F) is important data for each subject in the study.

## 5. Implementation of POA in this study

### 5.1. Course background

The first author teaches an integrated English course which aims at developing students’ English proficiency in English writing, listening, and speaking aspects. This study focuses on the speaking part in the course. The theme for the speaking task is *Boosting Culture Confidence*, and the teaching objectives are threefold as shown in Table 3.

**Table 3:** Teaching objectives.

a. Knowledge level	Discuss why we should have culture confidence and what measures have been taken to boost it
b. Skill level	Identify the intended audience of a speech and justify your answer
c. Cognitive level	Make a speech according to your audience’s characteristics

While the first teaching objective is at the knowledge level, which is easier to achieve with enough input like reading and listening, the second objective is at the skills level, and the third objective is at the cognitive level. This echoes the main principles in POA, and we focus on the teaching goal of “Identify the intended audience of a speech and justify your answer” in the current study.

### 5.2. Teaching process

#### 5.2.1. Teaching Cycle 1 using the traditional intensive reading approach

The task in this cycle is to introduce the mascots in the 19th Asian Games to different foreigners in English. Table 4 shows the first teaching cycle in a traditional method of IRA: lecture on the content and skills first with assignments and assessment followed, in other words, input comes before output.

**Table 4:** Cycle 1.

Procedure and actions	
Lecture	Share the design idea of the three mascots in the 19th Asian Games from the official website, and provide a word bank of useful expressions
Assignment	Present the scenario for and ask students to compose the script of an introduction for foreigners when working as a volunteer in the upcoming event
Assessment	Graded by machine (FiF) + teacher
**Results from Cycle 1**	
Test results	Average score of students’ production: 6.2 The students’ production shows homogeneity affected by the information from the official website. (TR1-G)
Student interview	“Task is not challenging” (SI1-1), “We know the mascots” (SI1-3), “The content information is ready online” (SI1-3), “I think it’s easier to put it down in written form than actually speaking to the foreigners” (SI1-4). “I’m not 100% sure (whether) foreigners they could understand what I’ve told them” (SI1-6)
Observation	Students’ engagement was low during the lecture part
Teacher reflection	Students’ production lacks concern for different audiences. (TR1-G) Students’ production shows homogeneity affected by the information from the official website. (TR1-G)

The researchers found a problem in the homogeneity in students’ output, especially in terms of content and style, with an overlap of nearly 70% seen from the sentences taken from the official website of the 19th Asian Games. Moreover, the students were less confident if “asked to introduce the mascot to foreigners in oral form” as “reciting the script does not seem natural” (SI-4). After reflection and discussion with colleagues, the researchers decided to adopt a different teaching approach to see how to improve students’ production.

#### 5.2.2. Teaching cycles using the POA

Based on the reflection and discussions with colleagues, together with the teacher professional development (TPD) training experience on the POA during the 6-week winter holiday, the researchers decide to adopt the POA of starting teaching at the beginning of the new semester. The teaching procedures in this study fall into three phases: driving, enabling, and assessing. Table 5 presents the POA teaching actions and the results in the second cycle, and Table 6 presents the POA teaching actions and the results in the third cycle.

**Table 5:** Cycle 2.

Procedure and actions	
Driving	Present the scenario and ask students to compose the script of introduction to foreigners when working as a volunteer in the upcoming event
Enabling	Share the link to the official website for the mascot in the 19th Asian Games (language enabler + content enabler) + lecture on audience analysis (skills and Structure enabler)
Assessing	Present a sample of introducing mascot Chenchen and analyze the key points of audience awareness and cultural concern Assessed by machine (FiF) + Students’ collaborative assessment
**Results from Cycle 2**	
Test results	Average score of trial production: 6.1 Average score of final production: 7.8
Student interview	“I like the task” (SI2-2), “I think it is easy because the design idea in Chinese is available online” (SI2-2),” “I’m not sure whether I should write in an official tone or should I learn a lot from audience analysis and be more friendly?” (SI2-3) “The task is challenging revealed from the assessment part, and I’m a little confused” (SI2-5)
Teacher reflection	Students’ trial production shows homogeneity of content and lacks concern for different audiences (TR2-G) Students’ final output exposes a lack of cultural concern of the target audiences (TR2-G)

**Table 6:** Cycle 3.

Procedure and actions	
Driving	Present the scenario and ask students to compose a dialog on introducing the mascot to a certain group of foreigners when working as a volunteer in the 19th Asian Games
Enabling	Share the link to the official website for the mascot in the 19th Asian Games (language enabler + content enabler) + lecture on audience analysis (skill enabler) + present a sample introducing the mascot Chenchen and analyze the key points in audience awareness and mutual respect in culture concern (content and structure enabler)
Assessing	Teacher–student collaborative assessment (TSCA) done by sharing two students’ production: one is graded 88 and the other 70. Review mainly the key points of the introduction: interactive language and mutual respect of culture In-class and after-class assessment via TSCA assessed by machine (FiF) + teacher + peer students
**Results from Cycle 3**	
Test results	Average score of trial production: 6.5 Average score of final production: 8.0 Students’ trial production shows homogeneity affected by information from official website (TR3-G)
Student interview	“The task is interesting” (SI3-6), “I thought it was easy because we could use the information ready on line” (SI3-2),” “Seriously, I want to be a volunteer in the games” (SI3-4), “I cannot wait to try it out with the foreign audience” (SI3-8)
Teacher reflection	Students’ production shows homogeneity in Cycle 1 affected by the information from the official website (TR3-G) Students’ output at driving phase in Cycle 3 shows a similar problem like those in Cycle 1 and Cycle 2, homogeneity (TR3-G) The following production witnessed an upsurging preference of either describing the mascot or choose the audience in the sample (TR3-W) TSCA was conducted with collaboration in both establishing criteria and peer assessment in class regarding the two foci: language and culture (TR3-G)

The trial production at the driving phase in this cycle also showed homogeneity like that in Cycle 1 because of the content information from the official website of Asian Games as well as a lack of audience awareness. Therefore, the enabling phase was designed as a mini-lecture on audience analysis, and the teacher called students’ attention to the language style in the following production. Additionally, the teacher also narrowed the intended final product to the specific mascot Chenchen to a group of primary pupils from Egypt. At the assessing phase, a sample from the teacher was shown to facilitate students’ collaborative assessment. The final production presented a leap in language style but also “exposes a lack of culture concern of the target audience” (TR2-G).

The trial production in this cycle showed a similar degree of homogeneity to those in Cycle 1 and Cycle 2. Considering the “lack of culture concern of the target audience” (TR2-G), the teacher presented the sample of Chenchen in the enabling phase after the mini-lecture on audience analysis. This helps to show what was expected in the task and to navigate students so they get the gist of relating the cultural elements of both parties to show mutual respect for culture, thus arousing the audiences’ further interest. During the assessment phase, TSCA was strictly followed through the collaboration of both establishing criteria and peer assessment in class regarding the two foci of language and culture (TR3-G). The final production displayed some inclination of the students’ choices of writing about Chenchen, but overall, this indicates a sharp rise in the students’ cultural awareness of the task, attaining the goal of communicative skills.

#### 5.2.3. The design features of three phases in the POA

##### 5.2.3.1. Driving phase: careful design of the teaching scenario

The POA advocates for the teachers’ full preparation before, during, and after the teaching process. A well-designed driving activity is far more than warming up or providing motivation for activities in that it poses a real task for the students to complete. The challenging part at this stage is to design and present a proper scenario for the expected production from the students.

A proper scenario is to arouse the students’ interests in and enthusiasm for using English for communicative purposes. According to the POA, a good scenario should contain the following four elements: a suitable and interesting topic that might happen soon, a purpose of language communication, a clearly defined identity, and the designated occasion (Wen, 2015a). The driving activity is both a challenge and a test to expose the gap between the students’ knowledge and the knowledge necessary to finish the expected unit goal. Firstly, students are asked to try the productive tasks, putting themselves in the scenario. Then, the teacher makes explicit the learning objectives and requirements of specific tasks, leaving the students eager to learn more about the topic (Wen, 2015b). According to the POA, the scenarios could simulate the authentic situations students might encounter in their future work or studies. The scenario in this study is set in the context of the 19th Asian Games, when university students are encouraged to participate in the events as volunteers. The design for this language class is attractive to students since the “participation of events could bring more chances of practicing oral English and this in-class task is useful in preparing us for it” (SI1-3) and “This is a good way to spread Chinese culture to help foreigners know better about China today” (SI3-2).

###### 5.2.3.1.1. Scenario

As a volunteer at the 19th Asian Games in Hangzhou, you are to introduce to the visitors the three mascots, namely *Congcong*, *Lianlian*, and *Chenchen*. The visitors might be players who have just finished their game races or scholars who are interested in Chinese history, even a group of kids coming from Egypt. Please write a script of 120 words how you are going to introduce one of the mascots to your possible visitors. Choose one of the visitors/one group of the visitors, and introduce any of the mascot to them.

##### 5.2.3.2. Enabling phase: alignment, gradualness, and variety

The enabling phase is important as it provides necessary scaffolding for students to complete the expected task, integrating learning with use closely. In this stage, teachers describe the productive tasks, provide scaffolding for students, and guide them to complete the productive tasks step by step. The provision of the input materials and output activities as enabling activities is called an enabler: an enabler of language, enabler of content, and enabler of structure (Qiu, 2017). Therefore, the unit goal breaks down into several minor goals so that the teaching activities deal with them one by one. One point worth mentioning is that the mini output within the process is also regarded as an enabler because it is part of the process which is not the final product to be assessed. Furthermore, the enabling activities should be designed according to students’ present level of language proficiency to help them become more competent in finishing the expected productive task (Qiu, 2017). This design of assisting activities echoes the idea introduced by zones of proximal development theory where an individual gets scaffolding from experts and accomplishes the learning task.

The design of enabling activities abides by the criteria of alignment, gradualness, and variety (Qiu, 2020). The major steps in the current study involve the enabling of content (information about the three mascots), enabling of skills (audience analysis), and enabling of structure (awareness of mutual learning among civilizations). When reviewing the students’ trial productions transferred to texts, the teacher found that apart from the sentences from the official website, students showed little effort in introducing the mascot themselves. This indicates a lack of support in the language, so the teacher provided scaffolding here by uploading a list of words and expressions necessary for the description online and sentence pattern drill exercises in class. Regarding the problem of content and structure, the teacher designed a mini-lecture on audience analysis, facilitating the students’ comprehension of the expected task of introducing one of the three mascots to different groups of foreign visitors, emphasizing the differences in interaction between presenting and introducing. After that, students were encouraged to do selective learning based on the provided material and their own innovation. The result from the second production in Cycle 2 showed that most students added greetings in the beginning and some expressions of goodwill in the introduction, trying to be more interactive with the audience. This was better than the first production, but it presented a problem of superficial understanding of being interactive in the task. Rather, in Cycle 3 the teacher presented a sample in the enabling phase focusing on the exploration of counterpart culture as shared knowledge to show mutual respect for the culture, creating a “dialog” perspective to enhance the effect of the expected introduction task. In this way, the whole task was divided into several smaller sections with a specific learning objective accomplished in different teaching steps, which could be rearranged and repeated depending on the pedagogical needs until the students accomplish the productive tasks. Scaffolding in this phase embodies alignment with the learning goal and demonstrates gradualness and variety.

##### 5.2.3.3. Assessing phase: assessment for learning and assessment as learning

For teacher-student collaborative assessment (TSCA), the assessing phase in the POA attaches importance to the evaluation of the students’ productions. Instead of the traditional summative assessment and formative assessment, the POA avails both teacher and students of opportunities to identify and discuss each part of the students’ production for continual improvement. The POA advocates for teacher-student collaborative assessment on the basis that students can make better achievements through learning the criteria and applying it to evaluate their own works (Sun, 2017). This collaboration prioritizes teacher’s preparation and mediation in each step because a guideline and a set of rubrics are prerequisites for students’ participation in the assessment, ensuring there are agreed-upon criteria for the collaborative work.

In the current study, assessment during Cycle 1 simply invited the students to read their classmates’ work and grade them based on their overall impression of the quality, which was summative and subjective. Assessment during Cycle 2 tried to adopt teacher-student collaborative assessment in the POA by sharing a sample production written by the teacher and guiding the class to learn about the criteria before asking the students to read and grade their peers’ work. Students claimed in the interview that they felt competent grading their classmates’ work because they could “compare them with the teacher’s sample” (SIZ-R). However, in the teacher’s reflection journal, she admitted this “wasn’t a perfect TSCA,” as seen from her doubts: “Was every student clear about the criteria? What are the practical steps in grading their peers’ work? Is this fair for the assessed students?” “Could they assess other production different from this mascot?” (TRJ 2-1). Therefore, in Cycle 3 the teacher selected two typical pieces of student work and identified evaluation foci before class, introducing the criteria in class followed by analyzing the two with the whole class. Meanwhile, a checklist was distributed to help ease the steps in the pair work of trial assessing in class. Through discussion with partners and the teacher, the grading criteria were then revised as necessary based on the collaborative work. Students “understood the criteria better” (SI3-Z) and assessed their peers’ work as collected from the online platform “FiF” after class. The teacher’s assessment (taking up 80% of the final grade) came when all the students had finished their assessment (taking up 20% of the final grade) process, as the teacher’s assessment enjoys higher authority and creditability.

Through teacher-student collaborative assessment, increased engagement took place in the classroom as students were “busy” (SIZ-G) with learning the criteria, evaluating, and discussing their reasons with peers in the trial grading assessment process. Regarding the constant feedback, the assessment phase assesses learning and is in essence a part of the learning process.

## 6. Findings, discussion, and implications

**Figure 2:**
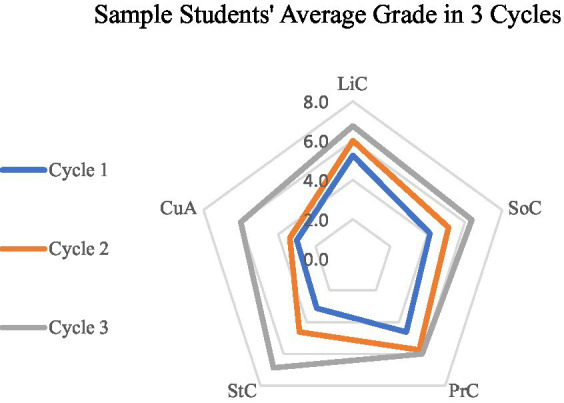
Comparison of sample of students’ average scores over three cycles.

Seen from the figure, students’ productions in the three teaching cycles result in better performance with the change in teaching method and continuous improvement of the teaching design (Figure 2). It shows the students’ major progress in aspects of Sociolinguistic Competence (SoC), Pragmatic Competence (PrC), and Strategic Competence (StC), but there were only minor changes in Linguistic Competence (LiC) and Cultural Awareness (CuA). The reason for this may be the topic of the Asian Games where students could get much information online, and were thus well-supported in language, and a lack of knowledge about foreign cultures because of their slow adjustment from learning in class to a self-learning mode.

The qualitative data are also echoed in the quantitative analysis despite the relatively small sample size (Table 7).

**Table 7:** The paired sample *t*-test.

The paired sample *t*-test						
		Paired differences		*t*	df	Sig. (two-tailed)
		Average value	Standard deviation			
Pair 1	C2/C1	0.96	0.86	3.17	7	0.016
Pair 2	C3/C2	1.71	0.85	5.72	7	0.001

The paired sample *t*-test reveals that the sample mean increased by 0.96 after the second cycle of action and increased by 1.71 after the third cycle of action. At a significance level of α = 0.05, the *p*-values for the first cycle/s cycle and second cycle/third cycle are both smaller than α. This suggests that there are significant differences in sample means after each cycle of action, indicating a positive effect of each action cycle on the sample mean.

An analysis of variance or a *t*-test can study the relationship between categorical data and quantitative data. A *t*-test can study the differences between two groups of samples, while ANOVA can study multiple groups. Therefore, we used ANOVA to investigate whether the three variables are significant (Table 8).

**Table 8:** Analysis of variance results.

Item	Group	Sample	Average	Standard deviation	*F*	*p*
Score	C1	5	4.02	0.98	9.767	0.003^**^
	C2	5	4.98	1.04		
	C3	5	6.42	0.43		
	Sum	15	5.14	1.3		

According to the results of variance analysis in Table 1, different groups have different scores, showing a significance level of 0.01 (*F* = 9.767, *p* = 0.003), and the comparison results of the group mean scores also showed significant differences.

Both qualitative and quantitative analysis showed the effectiveness of the teaching action. This study finds that the POA is more effective and beneficial for the learners as the scenarios designed in the driving phase are those “would-be” settings for the students, making the learning goals clear, specific, and interesting for the students to comprehend and easily put into practice. This is seen from both the results of the questionnaire and student interview during both the second and the third cycles. We found that 85% students suggested that they “found it interesting to learn about knowledge facilitating life around,” and more than 40% of the students mentioned in the interview that they “have a clearer goal as to what to learn in academic class,” which promotes students’ engagement in class activities. This echoes previous findings on effects of the POA on EFL learners’ performance in China’s tertiary education (Zhang W. J., 2020). Hence, the driving phase serves as the primary motivation in the development of academic literacy in the language classroom.

**Figure 3:**
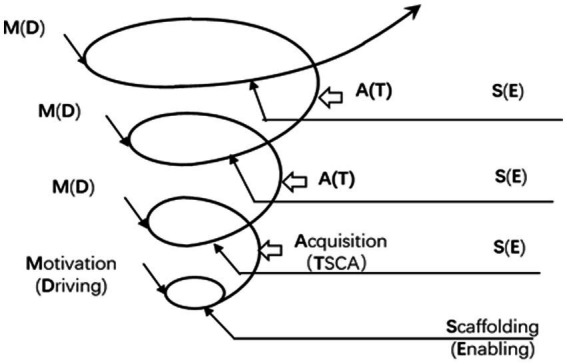
Mode of students’ acquisition of academic literacy.

The study also found that the design of the enabling activities helps to break down tasks into less challenging mini-tasks and provides students with necessary scaffolding to complete the task. The enabling activities’ features of alignment, gradualness, and variety embody the simultaneous integration of learning and the use of the principle of the POA. In this way, the teaching and learning efficiency rises, resulting in a greater sense of fulfillment for both the teachers and the students. Students become “aware of their role and responsibility in the learning process” (SI-07). This is an effective way to develop independent learning at the university level. A few students indicated that they became even “more confident in the interview of applying for a volunteer position in the coming Asian Games” (SIZ-H). This “scaffolding” is laudable in the academic literacy acquisition model (see Figure 3): all the enabling activities are well-designed, within the scope of the zone of proximal development. The enabling “scaffolding” is primary in the academic literacy acquisition model within the classroom learning context.

Findings also suggest that teacher–student collaborative assessment inspires students both in terms of enhancing their learning efficiency and in helping them acquire a new reflective learning mode. While the assessment phase was designed to promote teaching, the actual process proves that it is in itself a critical learning process. Based on our data, the original summative assessment turns into a formative assessment, triggering a series of self-learning activities, which to a great extent boosts the students’ sense of fulfillment. TSCA is not an end to the teaching and learning cycle because the assessment result during this phase serves as a trigger for a new round of exploration. Therefore, it is labeled as the “acquisition” phase in the academic literacy acquisition model.

Finally, the study also sheds light on the teachers’ professional development. The immediate implication is the application of a hybrid teaching model that ushers change in teaching pedagogy. In traditional language classes, teachers tend to assign listening and speaking tasks in the form of word bank completion or role play performances for students. However, within the framework of the POA, teachers feel a great necessity to equip themselves with new teaching methods to create authentic learning contexts to help with language learning and teaching. Even though the students’ English proficiency shows vital differences in each class, differentiated learning suggests the teachers can always rely on the POA model to design production-oriented driving, enabling, and assessment teaching sections as they are based on authentic language settings. Using a hybrid teaching mode also helps overcome the difficulty in teaching schedules outlined by the course syllabus, as the driving, enabling, and assessing procedures using the POA are production-oriented and are thus time-consuming. The teacher–student collaborative assessment makes it possible for students to reflect on their own performance against the suggested framework of assessment constructed by both parties. Instead of expecting a well prepared but homogenous output, students’ productions may vary as they are based on their unique results as compared with the sample and modified under the TSCA procedure, showcasing a differentiated learning mode. Furthermore, teachers’ awareness of conducting teaching following the POA procedure to keep the students more engaged helps their professional development in teaching, learning, and research.

## 7. Conclusion

There are diverse understandings and interpretations of academic literacies; it tends to become clearer that students’ competence in preparing themselves for academic settings depends on the quality and achievement of modern language education. The purpose of language learning is to conduct effective communication. Within the framework of the POA, the idea of learning driven by productive and meaningful tasks, the construction of authentic scenarios, and a collaborative assessment approach can all be viewed and justified considering sociocultural theory. Teachers should be aware of their students’ needs and learning habits nowadays and tailor their classes to suit their students’ interests and motivate them to engage in class activities; on the other hand, students should abandon the idea of finishing the course tests with only a passing grade and should instead incorporate the learning goal of their class.

The POA is tested and seen to be effective in developing EFL students’ academic literacy as they engage with authentic tasks. For many years in the Chinese EFL context, English language teaching and learning have been quite passive in the sense that language teachers find themselves in a knowledge transmission position in which the students become passive receivers. Within a POA framework, however, language teachers are afforded more alternatives to design tasks as they gage the students’ current language proficiency. For EFL students, this language learning approach is effective in creating a welcoming and comfortable environment where learners are invited to participate in various tasks, which contributes to their cross-cultural competence.

While this study sheds light on the effectiveness of POA in facilitating EFL students’ academic literacy development, future studies may benefit from two caveats in the current study. Since this is mostly a small-scale study, future research may increase the sample population to make the result more generalized to other EFL settings. In addition, the duration of this action research implementation is 8 weeks, which may not be long enough to yield a satisfactory outcome. Apart from these two caveats, studies could also explore identity issues as language teachers and students engage with academic literacy practices in class and more qualitatively oriented investigations of language teachers’ perceptions of pedagogical approaches in teaching academic literacy.

## Data availability statement

The raw data supporting the conclusions of this article will be made available by the authors, without undue reservation.

## Ethics statement

Ethical review and approval was not required for the study on human participants in accordance with the local legislation and institutional requirements. The patients/participants provided their written informed consent to participate in this study.

## Author contributions

YG: designing the research models, conducting the research, collecting and analyzing data, and writing the initial draft. HW: oversight and leadership responsibility for the research activity planning and execution, including mentorship, and review and editing. All authors contributed to the article and approved the submitted version.

## Funding

This work was supported by the Education and Teaching Reform and Innovation Program at Zhejiang International Studies University (2022).

## Conflict of interest

The authors declare that the research was conducted in the absence of any commercial or financial relationships that could be construed as a potential conflict of interest.

## Publisher’s note

All claims expressed in this article are solely those of the authors and do not necessarily represent those of their affiliated organizations, or those of the publisher, the editors and the reviewers. Any product that may be evaluated in this article, or claim that may be made by its manufacturer, is not guaranteed or endorsed by the publisher.
